# Super-resolution imaging with Pontamine Fast Scarlet 4BS enables direct visualization of cellulose orientation and cell connection architecture in onion epidermis cells

**DOI:** 10.1186/1471-2229-13-226

**Published:** 2013-12-28

**Authors:** Johannes Liesche, Iwona Ziomkiewicz, Alexander Schulz

**Affiliations:** 1 Department of Plant and Environmental Sciences, University of Copenhagen, Thorvaldsensvej 40, Frederiksberg 1871, Denmark

**Keywords:** Fluorescent dye, Cell wall, Cellulose, STORM, Structured illumination, Super-resolution microscopy, TIRF, Deconvolution

## Abstract

**Background:**

In plants, a complex cell wall protects cells and defines their shape. Cellulose fibrils form a multilayered network inside the cell-wall matrix that plays a direct role in controlling cell expansion. Resolving the structure of this network will allow us to comprehend the relationship of cellulose fibril orientation and growth.

The fluorescent dye Pontamine Fast Scarlet 4BS (PFS) was shown to stain cellulose with high specificity and could be used to visualize cellulose bundles in cell walls of Arabidopsis root epidermal cells with confocal microscopy. The resolution limit of confocal microscopy of some 200 nm in *xy* and 550 nm in *z* for green light, restricts the direct visualization of cellulose to relatively large bundles, whereas the structure of cellulose microfibrils with their diameter below 10 nm remains unresolved. Over the last decade, several so-called super-resolution microscopy approaches have been developed; in this paper we explore the potential of such approaches for the direct visualization of cellulose.

**Results:**

To ensure optimal imaging we determined the spectral properties of PFS-stained tissue*.* PFS was found not to affect cell viability in the onion bulb scale epidermis. We present the first super-resolution images of cellulose bundles in the plant cell wall produced by direct stochastic optical reconstruction microscopy (dSTORM) in combination with total internal reflection fluorescence (TIRF) microscopy. Since TIRF limits observation to the cell surface, we tested as alternatives 3D-structured illumination microscopy (3D-SIM) and confocal microscopy, combined with image deconvolution. Both methods offer lower resolution than STORM, but enable 3D imaging. While 3D-SIM produced strong artifacts, deconvolution gave good results. The resolution was improved over conventional confocal microscopy and the approach could be used to demonstrate differences in fibril orientation in different layers of the cell wall as well as particular cellulose fortifications around plasmodesmata.

**Conclusions:**

Super-resolution light microscopy of PFS-stained cellulose fibrils is possible and the increased resolution over conventional approaches makes it a valuable tool for the investigation of the cell-wall structure. This is one step in method developments that will close the gap to more invasive techniques, such as atomic force and electron microscopy.

## Background

Cellulose microfibrils form the backbone of the complex cell wall of plant cells. The exact structure of the cellulose fibril network has important implications for our understanding of plant growth on a cellular level, as it has a direct role in controlling cell elongation [1-3]. In addition, precise knowledge of cell wall architecture facilitates the development of methods for efficient breakdown of plant cell walls in the production of biofuels [4,5].

Pontamine Fast Scarlet 4BS (PFS), a fluorescent dye that binds cellulose with high specificity, has been used to visualize bundles of cellulose bundles in the cell wall of Arabidopsis root epidermis cells [6]. With a confocal microscope, cellulose bundle orientation could be followed over time, providing the first direct proof of passive reorientation of cellulose bundles during cell elongation. Nevertheless, only the largest cellulose bundles could be visualized, as the resolution of conventional light microscopy is limited to around 250 nm in xy-direction. Cellulose microfibrils have a diameter below 10 nm [7,8], but can be bundled into macrofibrils. Destructive methods that offer resolution on the nm scale such as scanning electron microscopy and atomic force microscopy indicate that macrofibrils do not necessarily show the same orientation as the majority of microfibrils [9,10].

In order to achieve a more complete and dynamic understanding of the structure of the cellulose network in plant cell walls a resolution increase for light microscopy is required that allows showing the cell wall in its native state and its differentiation in living cells. The extensive preparation of invasive high resolution techniques, such as scanning electron microscopy (SEM) and atomic force microscopy (AFM) are not compatible with live microscopy and were discussed to have profound impact on the cellulose network structure [9,11].

Recent developments in the field of light microscopy significantly increased the resolving capacity of fluorescence microscopes following different approaches (reviewed by Schermelleh et al. [12]). Basically, these approaches improve resolution by circumventing the diffraction-limits of microscopes as defined by Ernst Abbe [13]. We used two of these so-called super-resolution microscopy techniques, 3D-structured illumination microscopy (3D-SIM) and direct stochastic optical reconstruction microscopy (dSTORM), to test visualization of PFS-stained cellulose fibrils. The third of the established super-resolution techniques, stimulated emission depletion microscopy (STED) is not compatible with green plant tissues, since the depletion laser wavelength is highly absorbed by chlorophyll in the sample. In comparison to wide field fluorescence microscopes, 3D-SIM and STORM improve the resolution of fluorescent signals by, respectively, 2 fold in xyz and by up to 10 fold in xy [12]. In addition, we also tested the effects of deconvolution on confocal image stacks which leads to clearer micrographs by removing the distorting effects of the point-spread function. This function is an inherent factor of any optical system and can be compensated by appropriate algorithms.

Onion bulb scale epidermis, which is used in the present study, has been identified as an ideal model system for the investigation of how cells control growth [14]. Knowing the exact structure of the cellulose network in the cell wall is therefore of immediate significance.

## Results and discussion

### Spectral properties and cell toxicity of Pontamine Fast Scarlet in situ

**Figure 1:**
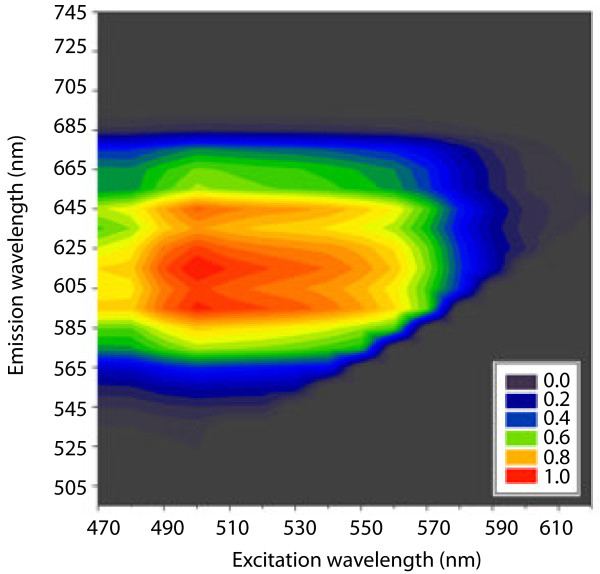
Contour plot showing the relative emission of onion epidermis cell walls stained with Pontamine Fast Scarlet 4BS (PFS) as a function of excitation and emission wavelength.

A simultaneous excitation and emission scan on PFS-stained onion bulb scale epidermis cell walls shows a broad excitation range from 485 to 565 nm (Figure 1). It is therefore well suited for use with the popular 488 nm and 561 nm laser lines. The results mirror earlier measurements performed *in vitro*[6,15]. The emission maximum was detected in the red spectrum with a broad peak around 615 nm (Figure 1). It can therefore readily be distinguished from GFP, YFP, FITC and other commonly used fluorophores that are excited at 488 nm. The partial overlap of the emission spectra of chlorophyll autofluorescence and PFS fluorescence is in most cases not a problem because chloroplasts and cell walls are easily distinguished.

**Figure 2:**
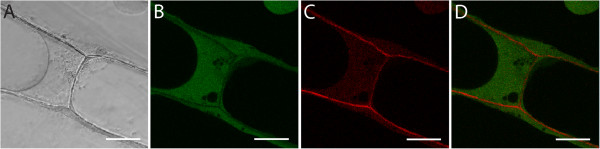
**Confocal micrographs of the onion bulb scale epidermis co-stained with PFS and Carboxyfluorescein-Diacetate (CFDA) for 4 h show non-toxicity of PFS. (A)** Bright field image. **(B)** CFDA fluorescence. **(C)** PFS fluorescence. **(D)** Overlay of **B** and **C**. Scale bars 50 μm.

The viability of the plant cells after staining with PFS was tested with carboxyfluorescein-diacetate (CFDA) [16]. The nonpolar CFDA molecules are able to pass the plasma membrane. In the cytoplasm, they gain their fluorescent properties through cleavage by endogenous esterases. Intact cells therefore show a green signal in the cytoplasm, where CFDA accumulates. The absence of esterase activity in dead cells prevents the activation of the fluorescence. In confocal microscopy (Figure 2), the CFDA signal could be observed in the cytoplasm of onion bulb scale epidermis cells even after 3 hours of staining with PFS. This did not change with longer incubation times (data not shown), demonstrating the potential of PFS as a general cell wall stain in long-term live-cell experiments. Propidium Iodide, the most common general cell wall dye with emission in the red spectrum, in contrast, is mutagenic, posing a health threat for sample and scientist [16].

### Direct stochastic optical reconstruction microscopy shows PFS-stained cellulose fibrils on the cell surface

**Figure 3:**
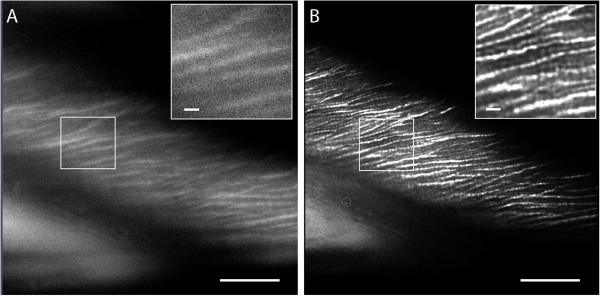
**Super-resolution images of PFS-stained cellulose fibrils in onion bulb scale epidermis cells. (A)** Total internal reflection fluorescence (TIRF) image. **(B)** Rendering of stochastic optical reconstruction microscopy (STORM) results. Scale bars 10 μm, 1 μm in inserts.

In direct STORM (dSTORM), fluorochromes can be switched between a fluorescing and a dark state when using an appropriate excitation wavelength and intensity. Due to the stochastic character of the fluorescent state, it is recorded as blinking and allows separation of neighboring fluorophore molecules in time. If a sufficient number of photons during single blinking event is recorded, the resolution can be improved down to 30 nm [17]. This method can be used most efficiently with photo-switchable fluorophores, but it has been shown that dSTORM is also possible with conventional dyes, based on their inherent switching capacity [18]. A pre-requisite for dSTORM is that the excitation is limited to a narrow optical section to exclude localization uncertainty that would be present in a large z-volume. This is generally achieved by total internal reflection (TIRF) microscopy. After PFS-staining, cellulose fibrils could be visualized in the cell walls of onion bulb scale epidermis cells using TIRF (Figure 3A). After bleaching with strong excitation laser light, it was furthermore possible to reduce the PFS fluorescence signal so far that single blinking events could be recorded. Figure 3B shows that the rendering of super-resolution images based on the localization results, yields a significant gain in resolution.

Fundamentally, two factors are important for a good dSTORM dye: 1) The number of photons detected per dye molecule. This will determine the precision by which the dye molecules are localized. 2) The on-off duty cycle. Dempsey et al. defines the on-off duty cycle as: "the fraction of time a fluorophore spends in the on state". Even with perfectly localized dye molecules the duty cycle will influence the resolution of structures [19]. In regard to these factors, PFS is not an ideal probe for dSTORM. The observed blinking events suggest that PFS, when bound to cellulose, generally has only a few switching cycles before irreversible photodestruction and has a long dark state, meaning that the duty cycle is very low. In addition, during the on-time of 5–10 ms only a relatively low amount of photons can be detected. In practice, this means that a large number of images have to be acquired in order to get a complete super-resolution image.

The method might be improved by employing an imaging buffer containing an oxygen-scavenging system and a primary thiol as reducing compound. Reducing conditions stabilize the off-state of fluorophores, thus preventing their irreversible photodestruction. At the same time, the buffer conditions induce spontaneous recovery of fluorophore molecules to their on-state. Optimization of thiol concentration and excitation laser intensity could lead to an increased number of localizations per frame and duty cycles. This should result in faster imaging and higher resolution [20].

The cellulose fibrils in Figure 3B have a diameter of around 100 nm with clear spacing between them and appear to branch at various positions. This structure on the cell surface can be significantly different from inner layers, which are generally expected to show narrower-spaced, more uniform fibrils [9]. Nevertheless, the observed form and pattern reflect the structural changes achieved during cell expansion and can, as such, give indications of how this process happens. The dSTORM images obtained here could point to the possibility of increased aggregation of fibrils during restructuring, although data from other cell-wall layers obtained with a similar method would be necessary for comparison.

### PFS is not suited for visualization of cellulose with 3D structured illumination

In 3D-SIM, patterned excitation light is used to take images at various phases, rotations and focus planes. From the resulting diffraction patterns, an image with higher resolution than wide-field images can be obtained via mathematical reconstruction in the Fourier space. Usually, this technique is well suited for imaging in plant tissue as it works with conventional probes and moderately thick specimens [21,22]. It was, however, not possible to resolve cellulose fibrils in the outer cell wall of onion bulb scale epidermis cells clearly. The Additional file 1: Figure S1 shows a comparison of the PFS-stained outer wall epidermis in wide field and 3D-SIM mode with two different noise filter settings. While standard noise filtering results in loss of any structural information, minimal noise filtering results in typical rotation-symmetric 3D-SIM artefacts as seen in the center of Additional file 1: Figure S1. Only small parts of the image reveal a fibril-like structure. It cannot be ruled out that strongly ordered cell wall macrofibrils from other cell types, like sclerenchyma fibers, would be resolved by 3D-SIM. However, discrimination of artefacts from real structures appears difficult - in any case for the example used here.

The effects of different filter settings indicate that the background signal level in PSF-stained onion cells is too high. In this respect the 3D-SIM mode behaves like a wide-field microscope rather than a confocal microscope, where out-of focus fluorescence is strongly reduced already under recording.

### Confocal imaging combined with image deconvolution can show the cellulose fibril network in 3D

While not usually considered as a super-resolution technique, image deconvolution, a mathematical operation to recover signal from blurring and noise, can also be used to enhance the resolution capacity of conventional microscopes beyond the diffraction limit. In comparison to STORM and 3D-SIM the gain in resolution is modest, but image deconvolution has the advantage of being applicable to confocal image data. This means that no special imaging hardware is required and that structures deep inside the specimen can be imaged due to the optical sectioning capacity of confocal microscopes.

Data can be acquired with point-scanning, as well as with spinning disc confocal systems. We used a spinning disc confocal, which offers a higher acquisition rate and lower photobleaching than a point confocal. After acquiring an image stack it is imported into adequate image processing software, of which the open source ImageJ and the commercial Huygens SVI are considered to give the best results [23]. The critical factor for deconvolving images is the point spread function, which can be determined experimentally or theoretically. We chose theoretical calculation as the determination of experimental point-spread functions can be unreliable for thick specimen, like whole-tissue samples [24]. Nevertheless, improvement in deconvolution efficiency might be realized if reference objects at the actual focal plane, for example fluorescent beads attached to the outside of a cell, are included during imaging and then used to calculate the sample-inherent point spread function.

**Figure 4:**
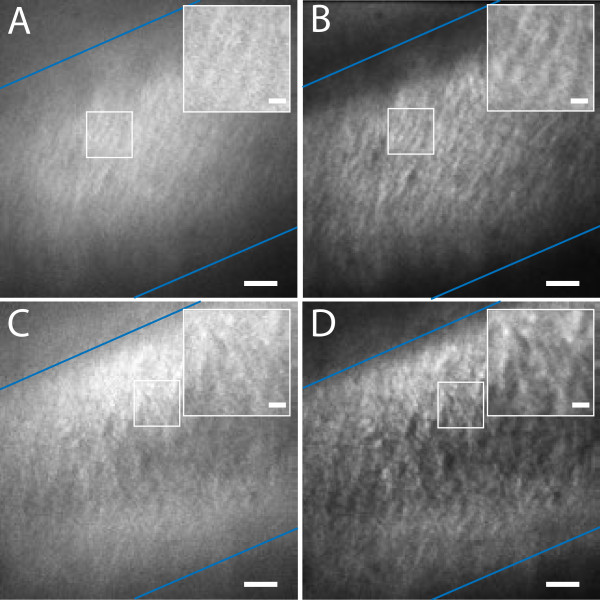
**PFS-stained cellulose fibril orientation imaged with conventional confocal microscopy (A, C) and in combination with post-acquisition image deconvolution (B, D); Cellulose fibrils are at an angle close to 45° at a focal plane about 100 nm below the outer cell wall surface (A, B) compared to an angle around 90° at a focal plane about 450 nm below the outer cell wall surface (C, D); Blue lines indicate borders to neighboring cells.** Scale bars 10 μm; 2 μm in inserts.

**Figure 5:**
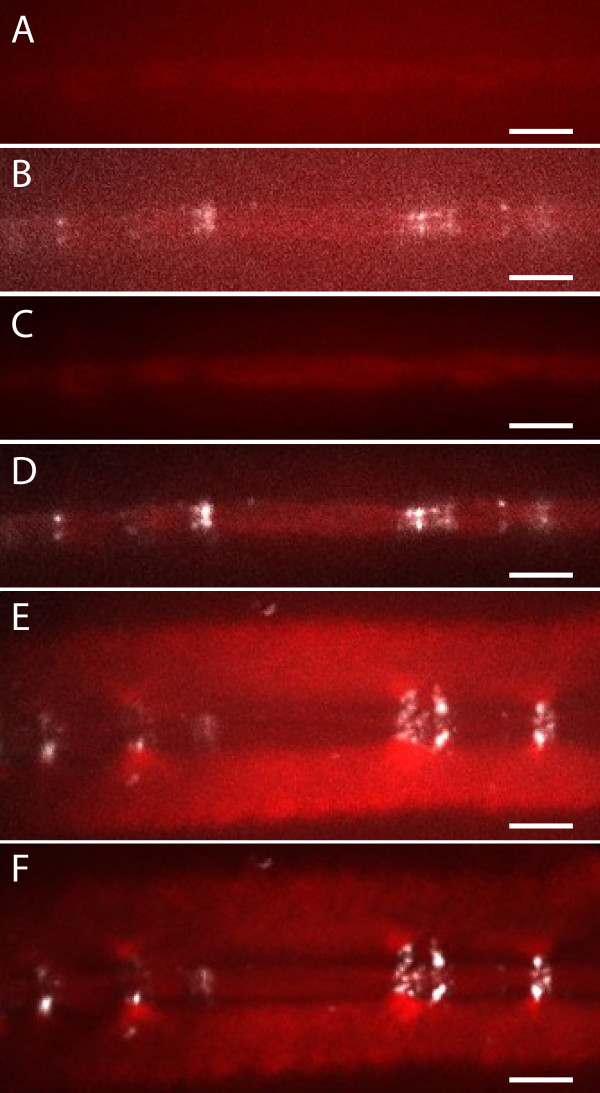
**Cellulose and callose around cell connections imaged with conventional confocal microscopy (A, B, E) and in combination with post-acquisition image deconvolution (C, D, F); a raw confocal image of PFS-stained cellulose in the cell wall between neighboring cells, b overlay with image of Anilin Blue-stained callose; After deconvolution the same images (C, D) show more clearly that callose and cellulose domains do not overlap; in maximum intensity projections a higher cellulose concentration can be clearly located around the callose depositions after deconvolution (F) compared to raw confocal images (E).** Scale bars 10 μm.

Application of deconvolution on image stacks of PFS-stained cellulose in the cell wall of onion epidermis cells improved image quality, providing a clearer view of cellulose fibril orientation (Figure 4) and cellulose depositions around plasmodesmata (Figure 5). The raw data shows that, similar to the situation in Arabidopsis root epidermis cells [6], the orientation of cellulose fibrils can be deduced from relatively large bundles, both at the outer (Figure 4A) as well as at the inner side (Figure 4C) of the cell wall. While the deconvolved images show principally the same fibrils, the enhanced resolution enables clear distinction of single fibrils (Figure 4B,D). This is essential for accurate quantification, for example of the angle between fibrils and the longitudinal axis of the cell. Measuring 20 fibrils, an average angle of 46.2° (standard error 3.1°) was determined at the focal plane 100 nm below the cell surface, while 20 fibrils 300 nm deeper inside the cell wall are arranged at n average angle of 92° (standard error 1.8°). Two distinct layers with different cellulose orientation reflects different cell expansion phases, in which cellulose in the outer layer reorients passively after xyz deposition as proposed in the passive reorientation hypothesis [25]. This information can complement onion epidermis growth studies that only analyze the mean cellulose orientation in the whole cell wall [3,14].

Taken the optical resolution of some 200 by 550 nm (x by z) with the objective lens applied (N.A = 1.4) at the emission maximum of PFS (600 nm), the observed change of fibril orientation at a focal distance of 200 nm appears impossible. However, the strength of deconvolution lies exactly in its power to improve the 3-D resolution by removing the blurring effects of the zeppelin-shaped point-spread function. This technique would not allow to separate two points in the specimen lying on top of each other on the optical axis (= *z*-axis) within this distance, but is able to do so for the linear structures as the macrofibrils in the sample used here. This means that two microfibrils running parallel on top of each other with a distance of 200 nm in *z* cannot be separated, but those which are crisscrossing. Here the deconvolution algorithm is able to improve the contrast between the fiber in focus and the gap (no fluorescence) 200 nm below sufficiently for visualization.

In addition to cellulose orientation, deconvolution of images of PFS-stained onion epidermis cell wall also reveals features of the cell connection architecture (Figure 5). While raw data of co-staining with the callose-specific dye Anilin Blue suggests incongruent distribution domains of cellulose in callose in the cell wall (Figure 5A,B), this becomes much clearer on deconvolved images (Figure 5C, D), even showing the thin cell wall plate that remains in the pit fields. Interestingly, in 3D view or maximum intensity projections of deconvolved images, it becomes apparent that cellulose is deposited at much higher density in areas of 2 μm around callose compared to the rest of the wall (Figure 5F). This cellulose collar could potentially form a limit to the callose deposits that are generally enhanced by wounding in order to plug the plasmodesmata [26].

## Conclusion

Wide-field fluorescence microscopy allows investigating cell walls in their native state, but further details of its substructure can be uncovered when improving the microscopic resolution power. Here we demonstrate that the cellulose-specific dye PFS is suited for use in TIRF microscopy and STORM, realizing the resolution of cellulose fibrils below 100 nm thickness. Nevertheless, since TIRF microscopy limits observation to the cell surface, no information can be gained from deeper cell wall layers. While 3D-SIM principally enables super-resolution imaging even more than 100 μm inside a biological specimen, the high signal density of PFS in the cell wall prevented a correct mathematical reconstruction of the fibrillar substructure in the onion epidermis. As an alternative to STORM and 3D-SIM we used conventional confocal microscopy with post-acquisition image deconvolution. This enables a more modest increase in resolution compared to the other approaches, but leverages the z-sectioning and deep imaging capacity of confocal microscopy. This approach yielded data that enabled us to assess the orientation of cellulose fibrils, which was different in two layers within the cell wall. It was also used in co-staining experiments with a dye for another cell wall component, which revealed new features of cell wall architecture around plasmodesmata between neighboring cells.

Future developments, like the adaptation of 3D-STORM [27] for plant tissues will, without doubt, help to further close the resolution gap between non-invasive light microscopy and invasive techniques like AFM and SEM. The results presented here show how a first step towards this goal can be realized with the super-resolution techniques that are currently available, demonstrating their potential to be a valuable tool for the investigation of cell wall structure of living plant cells at a resolution higher than what had been technically possible before.

## Methods

### Dyes and staining

Pontamine Fast Scarlet 4 BS was purchased from Aldrich Rare Chemicals Library (catalog no. S479896; Sigma-Aldrich, St. Louis, USA). It is now available under the synonym Direct Red. Stock solutions of 5 mg/ml in 0.1 M phosphate buffered saline (PBS) pH 7.2 can be stored at -4°C or -20°C for several weeks. The working solution was diluted to 0.1 – 0.01 mg/ml depending on the tissue and was always prepared fresh. Incubation time was usually 20 min, but longer in complex tissues. Staining was followed by brief washing in PBS.

Carboxyfluorescein-diacetate (Molecular Probes, Life Technologies Corporation, Carlsbad, USA) was used at 0.5 μg/ml in PBS. Tissue was stained in 10 to 15 minutes, followed by 5 minutes washing in phosphate buffer.

Aniline Blue (Sigma-Aldrich, St. Louis, USA) working solution was diluted to 50 μg/ml in PBS and staining time was 5 to 10 minutes.

### Sample preparation

Onion bulb scale epidermis was prepared from *Allium cepa* bulbs purchased in a supermarket. Using a razorblade 0.5 × 0.5 cm pieces were cut out from fresh scale and the epidermis peeled off from the inner side with tweezers.

### Spectral analysis

A simultaneous excitation and emission scan (Lambda^2^-scan) was performed *in situ* on onion bulb scale epidermis cell walls stained with PFS using a Leica SP5-X confocal laser-scanning microscope (Leica Microsystems, Mannheim, Germany). A white light laser, calibrated to keep a constant output power over the available spectrum, was used as excitation source. Excitation scans were made every 10 nm between 470 nm and 620 nm. The AOBS-defined detection window of 10 nm width was shifted from excitation wavelength plus 15 nm (center position) to 745 nm for each excitation wavelength. Image analysis was performed in ImageJ.

### Image acquisition and processing

For confocal imaging in the cell vitality test a Leica TCS SP2/MP (Leica Microsystems, Mannheim, Germany) was used. PFS and cFDA were excited by an Argon Laser at 488 nm. The following detection windows were defined: PFS: 560–605 nm, cFDA: 485–505 nm.

Super-resolution imaging was performed on a Zeiss Elyra PS.1 microscope (Zeiss, Jena, Germany) and an Andor iXion 860 EMCCD camera (Andor Technology, Belfast, Ireland) using a 561 nm solid state laser for excitation, a band pass filter with DM 561 nm, EM 570–650 nm.

For 3D-SIM 63 × 1.2 Zeiss Apochromat water-immersion and 63 × 1.4 Zeiss Apochromat oil-immersion objectives were used together with a 23 μm grating. Z-stacks were recorded with 5 phase-changes and 5 grating rotations for each section. The 3D-SIM wizard in the Zeiss Zen 2011 software was used for image processing with the following setting: SR frequency weighing 1, Baseline cut, theoretical PSF and noise filtering as specified in the image legend.

For STORM, the same microscope was used with a 100 × 1.46 Zeiss Apochromat oil-immersion objective. The TIRF angle was adjusted by focusing on the surface of the onion epidermis cells in epi-fluorescence mode, then switching to TIRF-mode and increasing the angle until cellulose fibers became visible (around 60°). For one image series, 20 000 images were acquired. These were processed inside the Zeiss Zen 2011 software using the PALM-wizard. The filter settings were chosen permissive compared to settings generally used when working with photoswitchable proteins with relatively low chi-square and background thresholds. False positives were filtered out by setting the minimum photon count to 100. From the vector map of localization precisions, the background-corrected point-spread functions were rendered with a localization precision of 75 nm. The on time and dark state interval of PFS fluorophores were determined in separate image series. Only a 128 × 128 pixel area of the camera sensor was read out in order to enable image acquisition times of about 3 ms per image.

Deconvolution of confocal images was done after recording optical sections with a spinning-disc confocal (Andor Revolution XD, 100 × 1.4 Olympus UPlanSApo objective, Andor iXion 897 EMCCD camera, 561 nm solid state laser, EX 564 nm, DM 561 nm, EM 570–650 nm). Deconvolution was performed in ImageJ using the plugins PSF Generator and DeconvolutionLab, both developed by Biomedical Imaging Group at the École Polytechnique de Lausanne [28]. With PSF Generator, theoretical point spread functions were generated with the Born and Wolf algorithm [29], which takes account of numerical aperture, refractive indices, wavelength, pixel size and z-step spacing. The resulting point-spread function was used in the deconvolution algorithm developed by Richardson and Lucy [30] with 20 iterations. This Bayesian-based method is generally considered among the most appropriate for deconvolution of confocal data [24]. The output data is compared to raw data without further image adjustments.

## Competing interests

The authors declare that they have no competing interests.

## Authors’ contributions

JL conceived and designed the study, performed experiments and wrote the manuscript. AS assisted with experiment design and discussed analysis and interpretation of image data and the manuscript. IZ assisted with interpretation of super-resolution image data and image processing. All authors read and approved the final manuscript.

## Supplementary Material

Additional file 1: Figure S1 3D-structured illumination of PFS-stained cellulose fibrils in onion bulb scale epidermis cells; On the wide-field image (**a**) a high background fluorescence intensity in relation to the in-focus area is obvious. On the same image after processing with standard noise filtering (**b**) all structural information is lost. Using minimal noise filtering instead (**c**), artifacts cover most of the in-focus area. Some high-resolution structural information might be preserved (box, same area magnified in insert). Scale bars 10 μm; 1 μm in insert. Click here for file
