# Supplementary figures and images for: Super-resolution imaging with Pontamine Fast Scarlet 4BS enables direct visualization of cellulose orientation and cell connection architecture in onion epidermis cells
Source: BMC Plant Biol. 2013 Dec 28;13:226. doi: 10.1186/1471-2229-13-226 (PMC3942175; doi:10.1186/1471-2229-13-226)

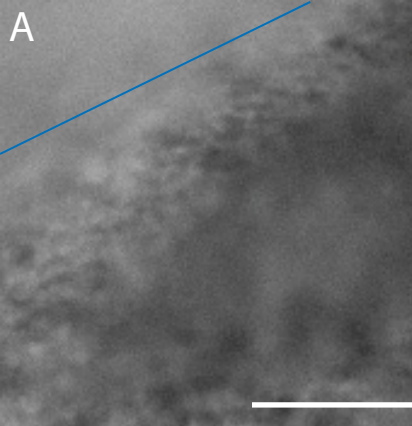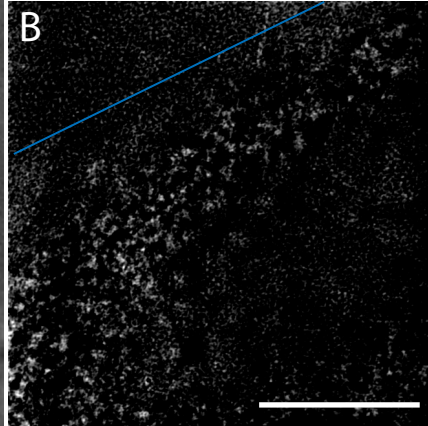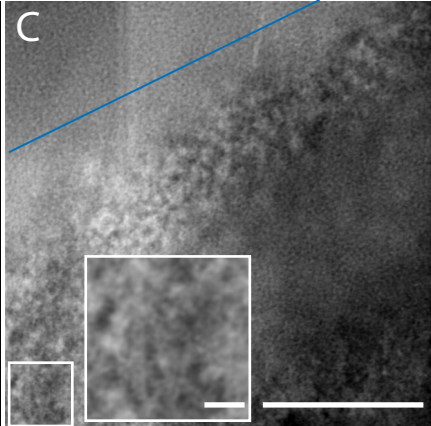

Supplement: Additional file 1: Figure S1 — 3D-structured illumination of PFS-stained cellulose fibrils in onion bulb scale epidermis cells; On the wide-field image (a) a high background fluorescence intensity in relation to the in-focus area is obvious. On the same image after processing with standard noise filtering (b) all structural information is lost. Using minimal noise filtering instead (c), artifacts cover most of the in-focus area. Some high-resolution structural information might be preserved (box, same area magnified in insert). Scale bars 10 μm; 1 μm in insert. [file 1471-2229-13-226-S1.pdf]
